# Matamatas *Chelus* spp. (Testudines, Chelidae) have a remarkable evolutionary history of sex chromosomes with a long-term stable XY microchromosome system

**DOI:** 10.1038/s41598-022-10782-z

**Published:** 2022-04-23

**Authors:** Patrik F. Viana, Eliana Feldberg, Fábio Hiroshi Takagui, Sabrina Menezes, Richard C. Vogt, Tariq Ezaz

**Affiliations:** 1 Coordenação de Biodiversidade, Laboratory of Animal Genetics, Instituto Nacional de Pesquisas da Amazônia, Av. André Araújo 2936, Petrópolis, Manaus, AM CEP: 69067-375 Brazil; 2 Animal Cytogenetics Laboratory, Department of General Biology, CCB, Londrina State University, Londrina, Brazil; 3 Coordenação de Biodiversidade, Centro de Estudos de Quelônios da Amazônia, Instituto Nacional de Pesquisas da Amazônia, Av. André Araújo 2936, Petrópolis, Manaus, AM CEP: 69067-375 Brazil; 4 Institute for Applied Ecology, Faculty of Science and Technology, University of Canberra, Canberra, ACT 12 2616 Australia

**Keywords:** Cell biology, Evolution, Genetics, Molecular biology, Biodiversity

## Abstract

The genus *Chelus*, commonly known as Matamata is one of the most emblematic and remarkable species among the Neotropical chelids. It is an Amazonian species with an extensive distribution throughout Negro/Orinoco and Amazonas River basins. Currently, two species are formally recognized: *Chelus orinocensis* and *Chelus fimbriata* and although it is still classified as "Least Concern" in the IUCN, the Matamatas are very appreciated and illegally sold in the international pet trade. Regardless, little is known regarding many aspects of its natural history. Chromosomal features for *Chelus*, for instance, are meagre and practically restricted to the description of the diploid number (2n = 50) for *Chelus fimbriata*, and its sex determining strategies are yet to be fully investigated. Here, we examined the karyotype of *Chelus fimbriata* and the newly described *Chelus orinocensis*, applying an extensive conventional and molecular cytogenetic approach. This allowed us to identify a genetic sex determining mechanism with a micro XY sex chromosome system in both species, a system that was likely present in their most common recent ancestor *Chelus colombiana*. Furthermore, the XY system found in *Chelus orinocensis* and *Chelus fimbriata*, as seen in other chelid species, recruited several repeat motifs, possibly prior to the split of South America and Australasian lineages, indicating that such system indeed dates back to the earliest lineages of Chelid species.

## Introduction

The side-necked turtles from the Chelidae family represent one of the three main living lineages that make up the Pleurodira suborder. They have origin in South America, with fossil records dating from Gondwanic events^1–3^. Several studies have shown that chelid turtles diversified even before the split of Gondwana^4–7^, suggesting that besides vicariant events, dispersal events also drove the diversification of these species in the beginning of the fragmentation of Gondwana^3^. Such events explain the current distribution of the approximately 60 extant species of Chelidae that are restricted to the Southern Hemisphere, occurring throughout Australasia and South America^8–11^.

The Amazon region is considered a *hotspot* and primary source of Neotropical biodiversity^12^, including for species of freshwater turtles^13,14^, with groups that are widely distributed along the Amazon hydrographic basins, such as turtles of the genera *Chelus* and *Mesoclemmys*^15,16^.

The genus *Chelus*, or simply Matamata, is clearly one of the most emblematic and charismatic species among the Neotropical chelids, being considered one of the most bizarre species in the world due to its particular lifestyle and unusual external morphology, such as triangular head, tiny eyes, extremely elongated neck and a long tubular nose. It is also among the largest species of the Chelidae family^17,18^. Besides, Matamatas are one of the few species in the world that are predominantly carnivorous, with a diet almost exclusively based on live fish^19,20^.

The Matamata, for centuries was considered as a monotypic species with a wide distribution, however, recently genetic and morphological analyzes revealed the existence of at least two different species occurring in different waterscapes along its extensive distribution in the Amazon region, namely: *Chelus orinocensis* and *Chelus fimbriata*^21^. *C. orinocensis* occurs primarily along the upper Negro and Orinoco River Basins, while *C. fimbriata* occurs in Solimões/Amazonas River Basin^16^ and both species date back to the late Miocene, with recent 13my of independent divergence^21^.

Despite the Matamatas are one of the most well-known species to the general public, especially due to its peculiar morphology and appreciation in the international pet trade^8,22^, many aspects of their natural history still remain unknown. Chromosomal features, for instance, are scant and practically limited to the description of the diploid number (2n = 50) for *Chelus fimbriata*^23–25^. Chelids stands out as the most diverse group among Pleurodira, exhibiting several karyotype configurations, varying including in number of macrochromosomes (*Mac*) and microchromosomes (*mic*), as well as the presence of differentiated and undifferentiated sex chromosomes^24,26–28^. Most species have the GSD (genetic sex determination) mechanism as mode of sex determination^29,30^, however, despite the evidence of one bivalent with no pairing in meiosis of *Chelus fimbriata*^23^, the sex determining strategies of Matamatas remain unanswered, as well as whether the species have sex chromosome systems, as observed in the vast majority of species in the family^27–29,31–33^. In this sense, a more detailed investigation into the karyotype composition of the two species of *Chelus* is required, especially to investigate the sex determination strategies and presence of *Mac* or *mic* sex chromosomes of this eccentric amazonian turtle species.

In this study, we aimed to provide a full description of the karyotype composition for *Chelus orinocensis* and *Chelus fimbriata* from the Amazon Rainforest. For that, we applied multiple conventional (Giemsa staining and C-banding) and molecular (Comparative Genomic Hybridization (CGH), FISH mapping of telomeric (TTAAGG)n, 18S rDNA and Simple Short Repeats (*SSRs*) sequences) cytogenetic tools. Also, we identified sex chromosomes and modes of sex determination in these charismatic turtles and discussed the evolution of sex chromosomes and the role of repetitive sequences along their diversification in the Amazon region.

## Results

### Karyotype and C-positive heterochromatin

**Figure 1:**
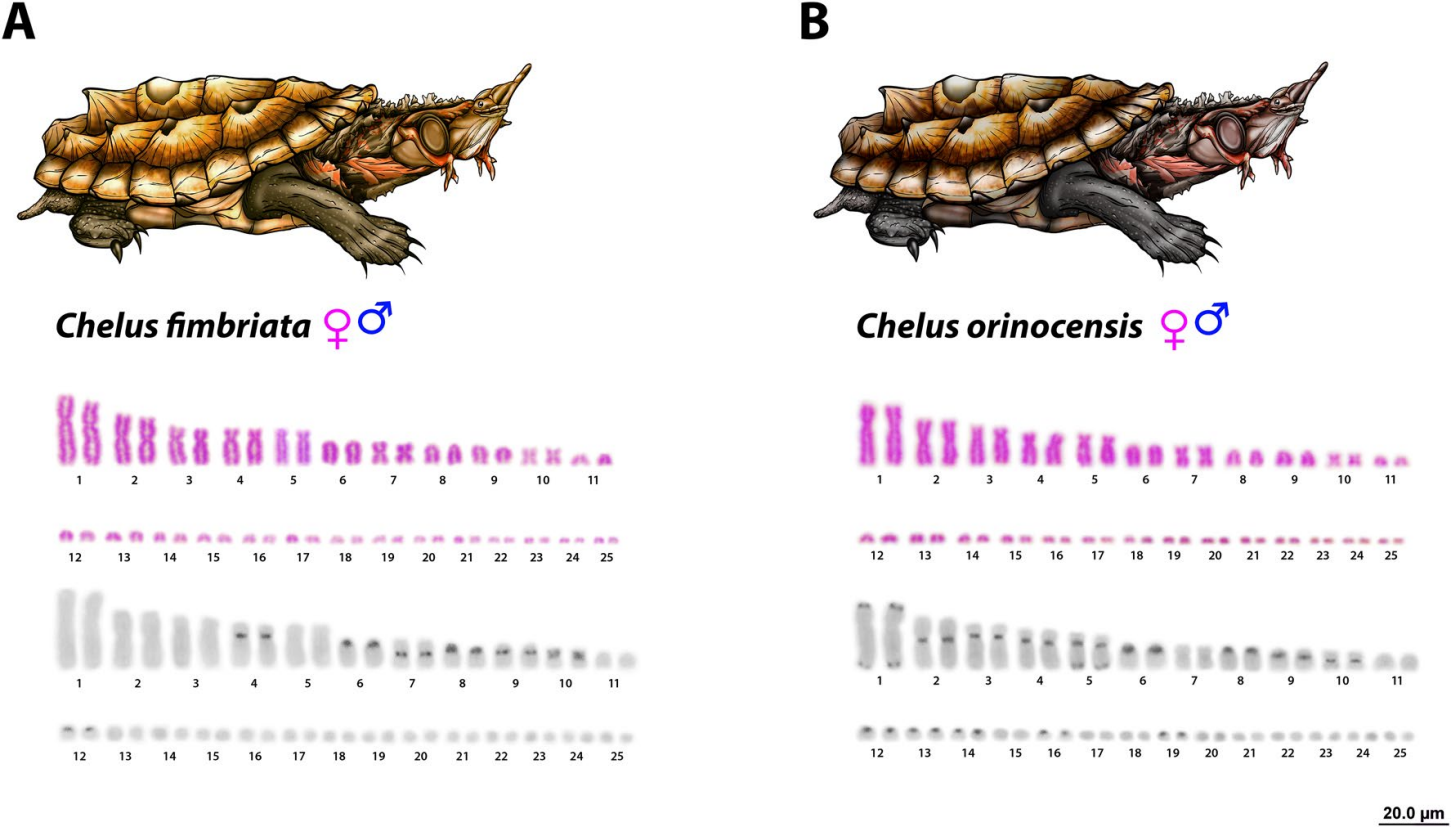
Karyotypes of males and females of *Chelus fimbriata* (**A**) and *Chelus orinocensis* (**B**) in Giemsa-staining and C-banding (**C**, **D**). Bar = 20 µm. Since male and female karyotypes are identical in morphology and in C-banding pattern, we selected a representative male metaphase to illustrate the results.

The Matamatas have 2n = 50 chromosomes with 22 *Mac* and 28 *mic*. The karyotype is composed by 6m + 4sm + 4st + 8a and 28mi (*mic* predominantly acrocentrics) for both species, with a fundamental number (NF) equal to 64 for males and females (Fig. 1). In *Chelus fimbriata*, the C-positive heterochromatin was found in few chromosome pairs (4, 6, 7, 8, 9, 10 and 12) (Fig. 1A), with a preferential accumulation on the centromeric regions, excepting the 10th pair, which had the short arm completely heterochromatic. In contrast, *Chelus orinocensis* exhibited a greater amount of C-positive heterochromatin (pairs 1–6, 8, 9, 10, 12, 13, 14, 16 and 19) (Fig. 1B), with conspicuous markings predominantly on centromeric position, but also on telomeric regions (pair 1) and on terminal position of the 5th chromosome pair. The *mic* pairs (12, 13, 14, 16 and 19) had only centromeric markings.

### Mapping of SSRs motifs, 18S rDNA and Telomeric repeats

**Figure 2:**
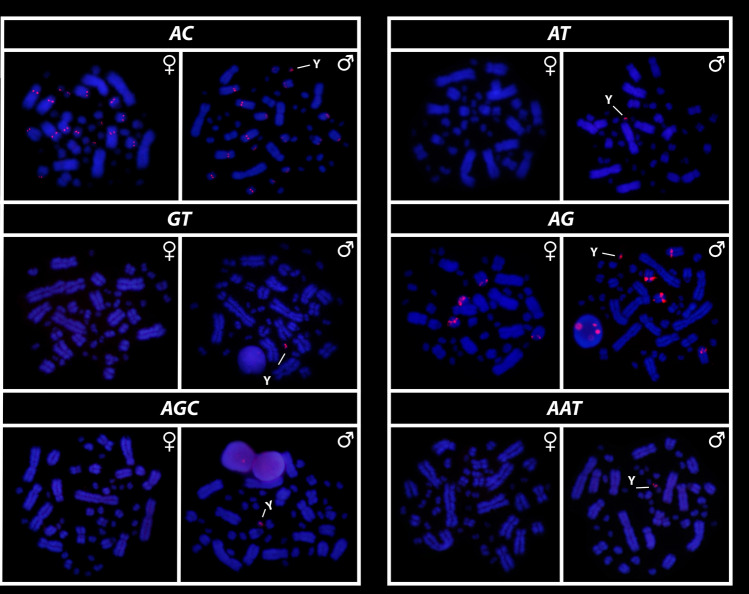
Mapping of several SSR repeats on the chromosomes of male and female of *Chelus fimbriata*, with highlights to an association of AG motif with the 9th pair that harbors the rDNA sites and the particular accumulation on the *mic* Y (lines).

**Figure 3:**
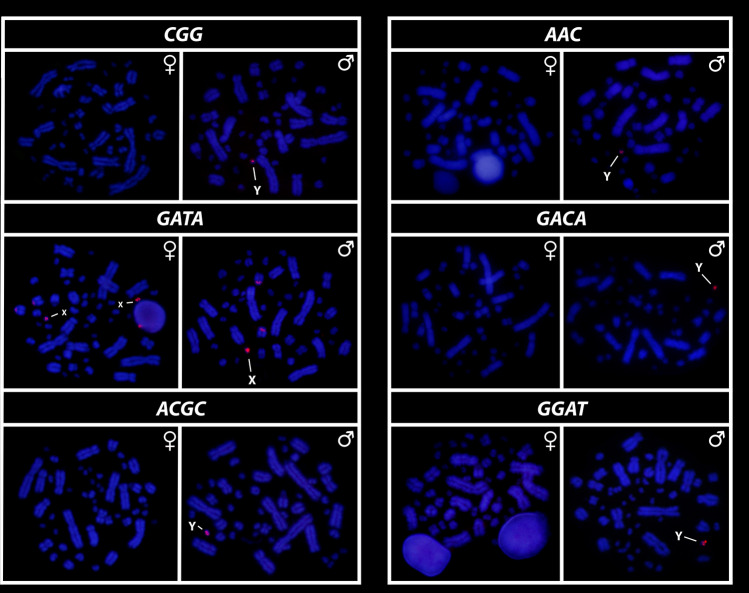
Mapping of tri and tetra-nucleotids on the chromosomes of male and female of *Chelus fimbriata*. Note the unique pattern of GATA motifs on X chromosomes (lines). The other SSRs showed only Y-specific accumulation (lines).

**Figure 4:**
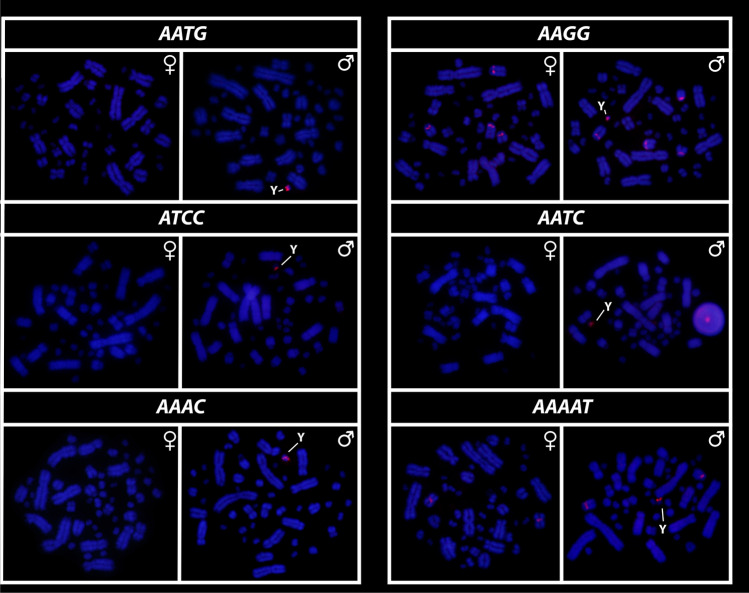
Mapping of SSRs on the chromosomes of males and females of *Chelus fimbriata*, with highlights to the particular accumulation on the *mic* Y (lines).

**Figure 5:**
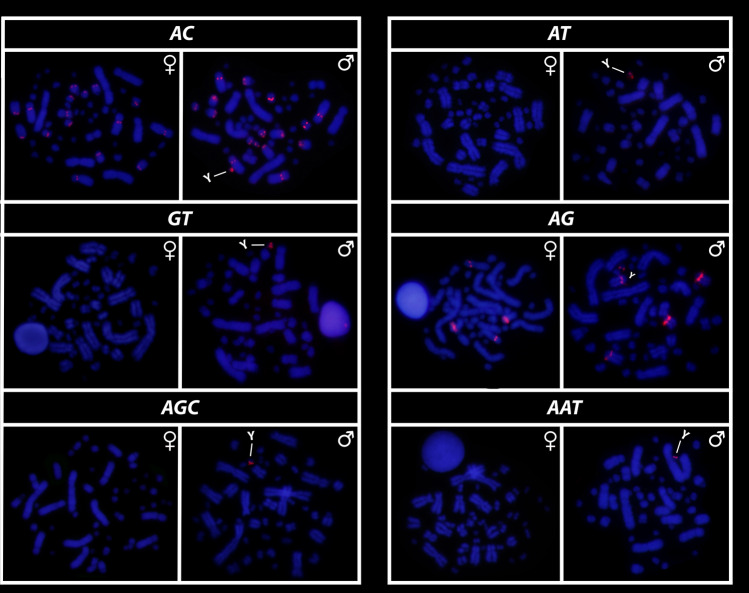
Mapping of several SSR repeats on the chromosomes of male and female of *Chelus orinocensis*, with highlights to an association of AG motif with the 9th pair that harbors the rDNA sites and the particular accumulation on the *mic* Y lines).

**Figure 6:**
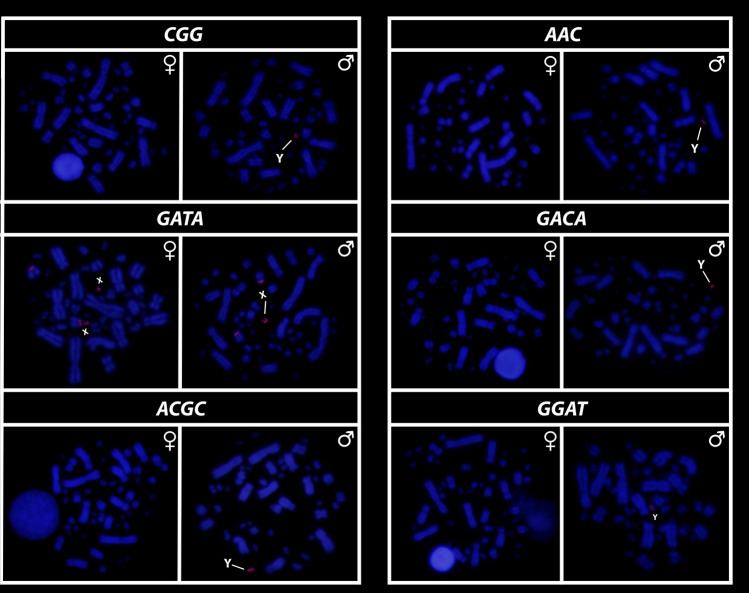
Mapping of tri and tetra-nucleotids on the chromosomes of males and females of *Chelus orinocensis*. Note the unique pattern of GATA motifs on X chromosomes (lines). The other SSRs showed only Y-specific accumulation (lines).

**Figure 7:**
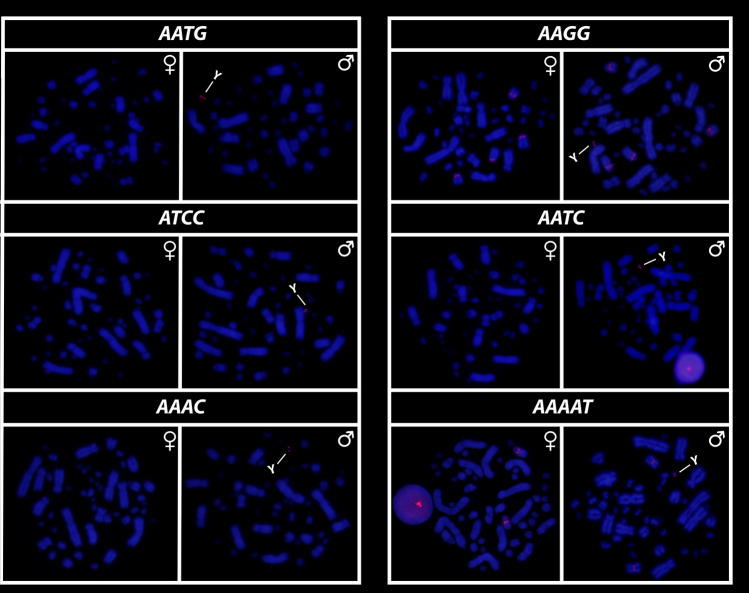
Mapping of SSRs on the chromosomes of males and females of *Chelus orinocensis*, with highlights to the particular accumulation on the *mic* Y (lines).

All eighteen microsatellite repeat motifs used (AC)_15_, (AT)_15_, (GT)_15_, (AG)_15,_ (AGC)_10,_ (AAT)_10,_ (CGG)_10,_ (AAC)_10,_ (GATA)_8_, (GACA)_8_, (ACGC)_8_, (GGAT)_8_, (AATG)_8_, (AAGG)_8,_ (ATCC)_8,_ (AATC)_8,_ (AAAC)_8_ and (AAAAT)_8_ showed hybridization signals on chromosomes of *Chelus orinocensis* and *Chelus fimbriata* (Figs. 2, 3, 4, 5, 6, 7). We found the same pattern of SSRs amplification for both species, including male-specific pattern of accumulation for some SSRs, which identifies *Chelus orinocensis* and *Chelus fimbriata* as a GSD species with a *mic* XY sex chromosome system and with X and Y-linked repeats (Figs. 2, 3, 4, 5, 6, 7). The (AC)_15_, (AG)_15,_ (GATA)_8_, (AAGG)_8_ and (AAAAT)_8_ showed hybridization signals on both *Mac* and *mic* of males and females, but with a particular amplification on the Y sex chromosome, excepting for the (GATA)_8,_ that was found to show a unique pattern (Figs. 3, 6), three markings in males (the 9th pair and a single *mic*) and four markings in females (the 9th pair and two *mics*), this reveals a particular amplification in females and identifies the X chromosome. On the other hand, the motifs (AT)_15_, (GT)_15_, (AGC)_10,_ (AAT)_10,_ (CGG)_10,_ (AAC)_10,_ (GACA)_8_, (ACGC)_8_, (GGAT)_8_, (AATG)_8_, (ATCC)_8,_ (AATC)_8_ and (AAAC)_8_ were found to be male-specific, with exclusive amplification (Figs. 2, 3, 4, 5, 6, 7). Besides, we were able to properly identify the X chromosome with specific accumulation of (GATA)_8_, even with the morphological similarity of the X with many other *mics*. Interestingly, even with particular accumulation of several SSR motifs, the Y chromosomes of *Chelus orinocensis* and *Chelus fimbriata* are not heterochromatic (Fig. 1), being identified solely through the mapping of the SSRs.

**Figure 8:**
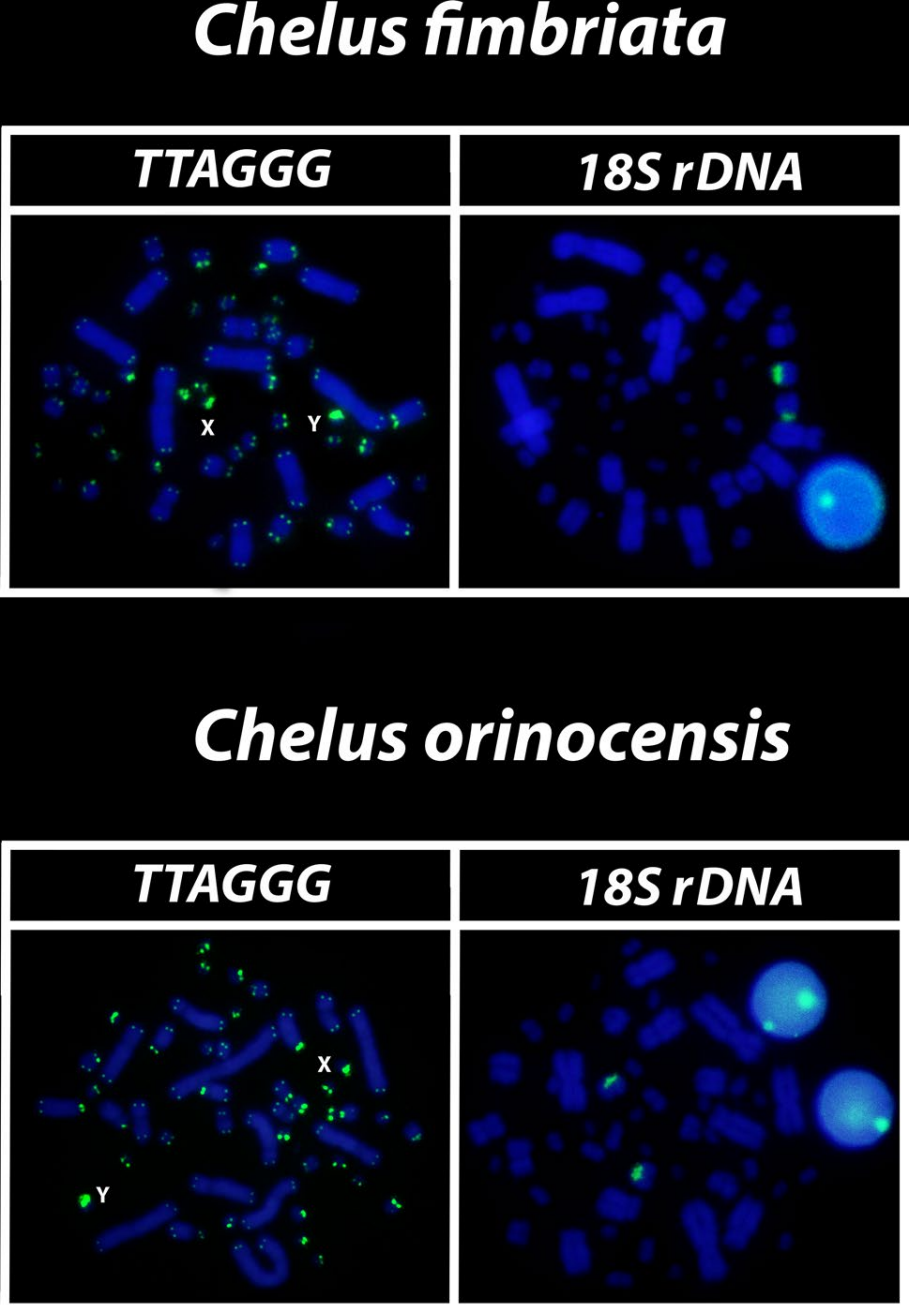
Mapping of telomeric motifs (TTAGGG)_n_ and 18S rDNA on the metaphase spreads of *Chelus fimbriata* and *Chelus orinocensis*. Please note the particular XY amplification of telomeric-like repeats in both species (letters).

The mapping of the 18S rDNA sequences showed simple markings on the secondary constriction of the second largest acrocentrics of the complement, the 8th pair in both species (Fig. 8). The telomeric motifs was evidenced on all terminal portions of all *Mac* and *mic*, with no evidence of ITSs (i.e., tandemly repeats of TTAGGGn sequence that are localized at intrachromosomal portions such as centromeres and interstitial positions and referred as Insterstitial Telomeric Sequences), nonetheless, a clear amplification of such sequences was evidenced on XY chromosomes (Fig. 8). The (TTAGGG)n amplification on X and Y was confirmed by the subsequently mapping of (GATA)n and (GACA)n respectively, in the same metaphase spread in males and females (picture not shown). Because the hybridization patterns of 18S rDNA and telomeric repeats for males and females were exactly the same, we selected a male representative metaphase to illustrate the results.

### Comparative genomic hybridization (CGH)

**Figure 9:**
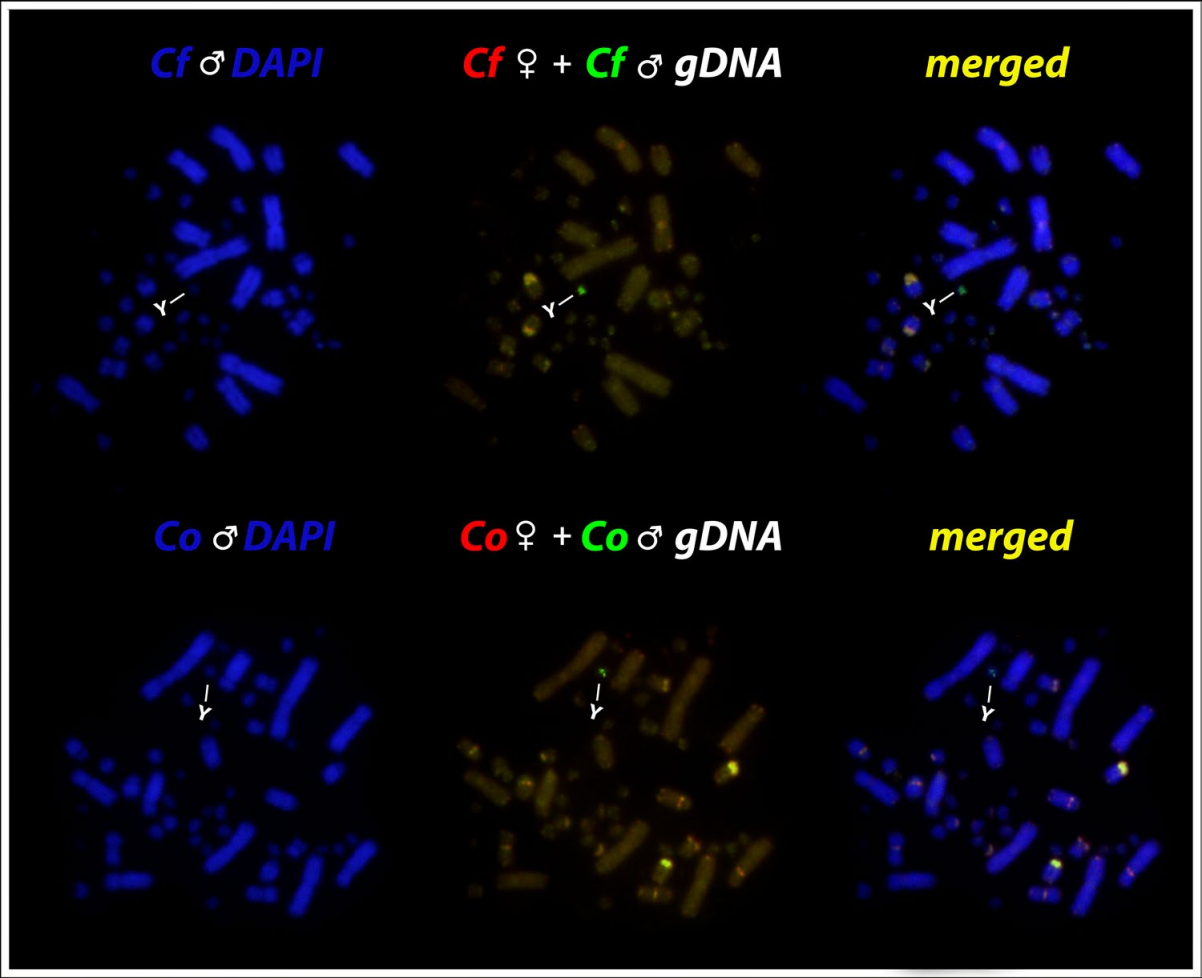
Chromosome spreads of *Chelus fimbriata* (Cf) and *Chelus orinocensis* (Co) males after CGH procedures using male (green) and female-derived (red) genomic probes. The common genomic regions are highlighted in yellow. Please note in green the male specific sequences Y-linked on a tiny *mic* (lines).

Our intraspecific comparison between males and females of *Chelus orinocensis* and *Chelus fimbriata* produced intense hybridization signals on centromeric position of some *Mac*, not necessarily collocated with heterochromatic portions (Fig. 9). Some *mic* showed scattered markings in both species, however, the merged images identified male-specific sequences accumulated in a tiny *mic* of the karyotype, the micro Y sex chromosome (Fig. 9). Since the hybridization signals on female metaphase spreads showed only shared sequences, we selected only male’s metaphase of *Chelus orinocensis* and *Chelus fimbriata* to illustrate the results and highlight the Y-linked markings. Sequential detection of C-positive heterochromatin in *Chelus orinocensis* and *Chelus fimbriata* in fact revealed that the Y chromosome in both species is not heterochromatic (image not shown).

## Discussion

In the present study, we cytogenetically analyzed the two extant species of Matamatas, the newly described *Chelus orinocensis* and reinvestigate the karyotypic composition of *Chelus fimbriata* since its first description almost half a century ago. We discuss hypotheses and trends on the evolution of sex chromosomes and the stability of the XY system that likely dates back to the origins of the Chelidae family ~ 103–160 Mya. Our findings corroborate previous mention in the literature regarding the karyotype composition of *Chelus*^23,24^. We also detected 2n = 50 chromosomes for the two species of Matamatas (Fig. 1), highlighting gross conservation of karyotypes between species, differing from each other only by the C-banding pattern (Fig. 1). Our study also provided evidence that both species have GSD with XY *mic* sex chromosomes.

In the late 70s, Barros and colleagues published the first karyotype description for the genus *Chelus*, (fairly similar to our findings), which had 2n = 50 chromosomes and no difference between males and females. However, the evidence of heterochromatic portions and one bivalent without pairing in meiosis already indicated that the species would be GSD with XY sex chromosomes system. Confirmation of the presence of sex chromosome in *Chelus* came just nearly half a century later with the findings from our study, where we evidenced a GSD mechanism as mode of sex determining and *mic* XY chromosomes in both analyzed species, similar to what has been described in Australian Chelid species.

**Figure 10:**
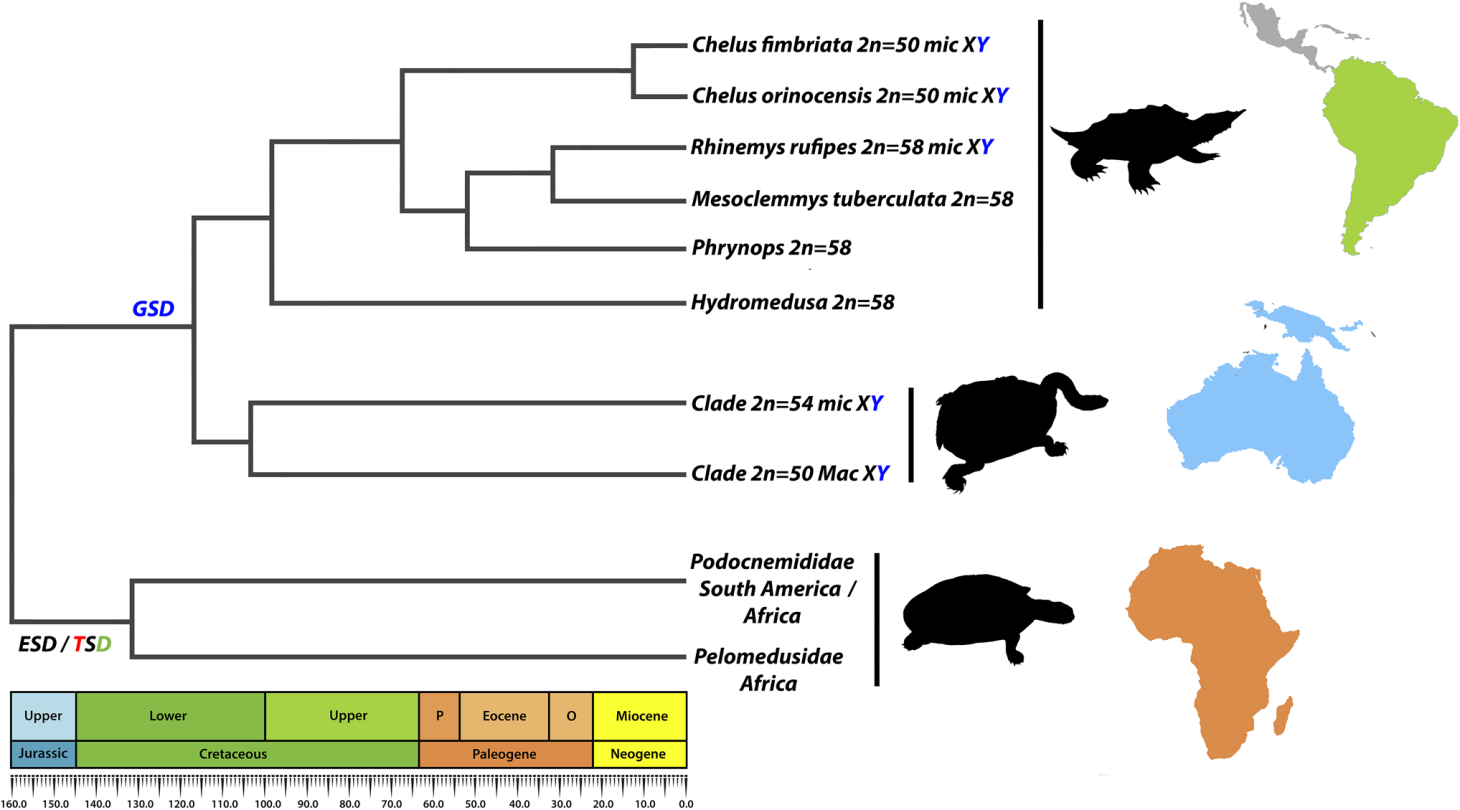
Chronogram for some chelid species and their modes of sex determination, adapted from^6,21^. P = Paleocene and O = Oligocene.

Modes of sex determining and sex chromosomes exhibit a striking evolutionary dynamic in vertebrates, with complex mechanisms that have evolved independently and multiple times across lineages and *tempo*^34–38^. Several reptile lineages follow the mainstream with multiple turnovers even in closely related groups^39,40^. Turtles also accompany this evolutionary path, with transitions among mechanisms of sex determining (Environmental sex determining—ESD/Genetic sex determining—GSD) and systems of sex chromosomes (XY/ZW) that evolved repeatedly along its evolutionary history^41–44^ and several intra/interchromosomal rearrangements are signed as the main source of such diversity^31,32,45–48^. In general, the GSD mechanism appears to be the sex determination mode chosen by the Neotropical and Australasian chelids since split from their sister families Podocnemididae and Pelomedusidae (both ESD/Temperature-dependent sex determination) (Fig. 10)^6,21,29,30,49^.

The ancestral state of *Mac* or *mic* sex chromosomes in Australasian chelids still remains as an open question^27,32,33,50^, although the most probabilistic scenario encompasses translocation/fusion events from *mic* to *Mac* sex chromosomes in the ancestor of the clades with *Mac* sex chromosomes (species with 2n = 50) and the clade with *mic* sex chromosomes (*Chelodina* spp. with 2n = 54)^32,33,48,50^. In Neotropical lineages, out of the three species analyzed using more refined cytogenetic tools, all have *mic* X [^28^, present study]. The *mic* XY of the Red Side-Necked Turtle *Rhinemys rufipes* dates back the Oligocene^28^ and much likely share the same origin with the XY of *Chelus orinocensis* and *Chelus fimbriata*, that goes back to the late Cretaceous/Paleocene^6,21^. The unquestionable South American origins of Chelidae^1–3^, and the likely ancestry of such *mic* sex chromosome in Neotropical lineages suggest that it was already present since the genesis of Chelidae in the Upper Jurassic ~ 103–160 Mya, highlighting an ancient and long-term XY system along the evolutionary history of these turtles. However, an overview of sex chromosome trends in a broader of neotropical chelids is still required to fulfill into this gap regarding the ancestor state in the family as a whole, as well as to uncover whether *Mac* or *mic* XY sex chromosome is present in the former Chelidae lineages. Test the homology of the known *mic* sex chromosomes of neotropical lineages (*Chelus* and *Rhinemys*) with those *Mac* and *mic* sex chromosomes of Australasia lineages is an essential step to highlight such ancestry.

The Chelidae chromosomes vary in number (including quantity of *Mac* and *mic*) and morphology of chromosomes^25,27,28^ and much of this diversity, as already mentioned, is attributed to several inter-intra chromosomal rearrangements that occurred along its evolution^32,48,50^. For example, the smallest metacentric pairs present in *Chelus orinocensis* and *Chelus fimbriata* (pairs 7 and 10) are missing in most Neotropical species with known karyotype^23,24,26,28^. With few exceptions (e.g., *Mesoclemmys gibba* 2 = 60, *Mesoclemmys raniceps* 2n = 40 and *Mesoclemmys* sp. 2n = 42), most Neotropical species have 2n = 58, where chromosomal fission events could be behind this diversity. If we consider centric fissions in the pairs 4, 5, 7 and 10 of the *Chelus* complement (2n = 50), this would result in exactly the same 2n = 58 and karyotypic formula seen in *Rhinemys rufipes*, *Mesoclemmys vanderhaegei*, *Mesoclemmys perplexa*, *Mesoclemmys tuberculata*, *Mesoclemmys dahli*, *Mesoclemmys hogei*, *Mesoclemmys nasuta*, *Phrynops geoffroanus* and *Hydromedusa tectifera* [^24,25,28,51,52^, personal data]. However, *Hydromedusa tectifera* (2n = 58) arose in the Middle Jurassic ~ 100 mya^6^, much older than 2n = 50 found in *Chelus* (Fig. 10). This raises two interesting and different evolutionary trajectories for chromosomal diversification of Neotropical chelids: (1) much older fusion events from the split of *Hydromedusa* and *Chelus* and, subsequently, fissions in the bi-armed pairs (4, 5, 7, 10) leading back to the 2n = 58 found in the most other neotropical chelids or (2), where the 2n = 58 would also represent the ancestral chromosome state and the 2n = 50 would represent a unique apomorphy arising solely in *Chelus* through fusion events from its most common recent ancestor after its split in the Middle Jurassic and the 2n = 58 was simply retained in most other Chelidae lineages. In fact, the 2n = 58 chromosomes seem to be the ancestral state for Chelidae, since the 2n = 50 appeared only twice along its evolution (in *Chelus* spp. with *mic* XY and in the Australasian lineages with *Mac* XY), possibly independently. Chromosomal paintings using the likely bi-armed chromosomes involved in these fusion/fission events is required to test the full homology across 2n = 50 and 2n = 58 chromosomes (as well as other 2n configurations in the family) and will certainly shed light on this evolutionary puzzle.

Despite these evident events of past chromosomal rearrangements orchestrating the karyotype diversification in Chelidae, with few exceptions^27,28,53^, relics of intra/interstitial telomeric-like repeats are rarely detected in turtles as a whole^54^. In *Chelus* spp. for example, no traits of ITSs have been found, but a particular amplification of telomeric-like sequences on XY chromosomes.

Amplification of telomeric repeats on sex chromosomes (XY and ZW) has been reported in a range of reptile lineages^50,55,56^, being frequently suggested as having regulatory activities and involved in heterochromatinization process. Telomeric-like repeats is clearly associated with *Mac* and *mic* sex chromosomes in Australasian chelids, including in a hybrid between *Emydura* and *Elseya*^27^. Clemente et al.^54^ mapped telomeric sequences in a male of *Mesoclemmys hogei* (South American lineage) and found no evidence of ITSs, but a tiny *mic* chromosome clearly showed a great accumulation of telomeric-like repeats, suggesting that this species might have an XY system, however, further analyzes with more males and females as well as the mapping of Y-linked SSRs (like those ones we have shown to be sex-specific in *Chelus* and in *Rhinemys*^28^ is necessary to confirm *Mesoclemmys hogei* as having *mic* XY chromosomes. In *Chelus orinocensis* and *Chelus fimbriata*, the amplification of telomeric-like repeats on XY, as well as the presence of several other SSRs sex-linked repeats apparently follow an alternative evolutionary path, not necessarily correlated to heterochromatinization process, since the XY in both species, unlike what was observed in *Rhinemys rufipes*^28^, were found to be non-heterochromatic. Perhaps, the recalcitrant recruitment of such repeats on these sex chromosomes might be related to a role beyond the regulation of the component of satellite DNA on XY, but also acting in the sex determining and in the maintenance of the dynamics of XY system architecture across Chelidae lineages.

Another repetitive and fundamental sequence, sometimes encountered in vertebrate sex chromosomes are the highly conserved rDNAs, which have a unique^57–61^ evolutionary dynamic, with multiple and crucial roles, acting from the control of cell aging/maintenance of genome integrity to shaping sex chromosomes, roles and functions far beyond ribosomal synthesis (for details on rDNA functioning, see Symonová^62^). In turtles, the 18S rDNA is frequently recruited on both ZW and XY systems^27,63,64^ and in Australasian chelids, such association is evidenced in both lineages with *mic* XY (2n = 58) and *Mac* XY (2n = 50)^27^. In Neotropical species, hitherto, no evidence of rDNAs accumulation on sex chromosomes has been found [^28^, present study] in addition, only *Mac* pairs harbor the rDNA sites in *Rhinemys rufipes* (pair 3), *Chelus orinocensis* and *Chelus fimbriata* (pair 8), all species with *mic* sex chromosomes. Seemingly, the accumulation of rDNAs on sex chromosomes is exclusive to Australasian species. Regardless, rDNAs are thought to somewhat represent an evolutionary driver for sex chromosome evolution in groups with which they are associated^60,65^, including Chelidae, where they have likely undergone multiple translocation events due to its association with other repetitive sequences (e.g., TEs/transposable elements and SSRs/simple short repeats), resulting in different chromosomal pairs carrying the rDNA. Interestingly, only one SSR motif (AG) was found bearing the 18S rDNA sites in *C. orinocensis* and *C. fimbriata* (Figs. 2, 5), while at least three (GT, AG, AAC) has been detected in *Rhinemys rufipes*^28^, probably reflective of different landscape of SSRs in these species.

SSRs represent an important and dynamic portion of genomes, that can evolve rapidly and independently^66–69^, being often associated with regulatory functions in genome architecture, structural activities in DNA, chromatin organization, gene expression and a myriad of other important functions^70–72^. This dynamic behavior, together with its expansion and contraction in genomes and high mutation rates remarkably reflects in distinct SSR landscapes, even in closely related species^69,73–78^.

Although turtles are naturally SSR-poor^68,69,79^, the frequent recruitment of such sequences to sex chromosomes indicates that SSRs repeats may be inherently shaping the evolution of sex chromosomes in chelids [^27,28,50^, present study] . The ATCC repeat motif, for example, is found on the sex chromosomes of at least 5 species of Chelidae [^28,32^, present sudy]. In Matamatas, thirteen SSRs are exclusively amplified on the tiny mic Y chromosome (Figs. 2, 3, 4, 5, 6, 7). Interestingly, six of them, found exclusively on sex chromosomes of *Chelus* spp. (GT, AAC, GACA, GGAT, ACGC, ATCC), are found in autosomes and on the *mic* Y of *Rhinemys rufipes* [^28^, present study], suggesting that in fact, closely related species may exhibit completely divergent SSR landscapes. In contrast, *Chelus orinocensis* and *Chelus fimbriata* shows exactly the same pattern of SSRs (Figs. 2, 3, 4, 5, 6, 7), which may be explained by their recent split (13Mya) and past genetic introgression and gene flow promoted by the wide distribution that these species had during the conformation of ancient Amazonian waterscapes over these million years of evolution^21^. This similarity of SSRs between the two Matamata species also suggests that the *mic* XY system could already be present in *Chelus colombiana*, the most recent common ancestor that gave rise to the two living species^80^.

Our study is the first to offer an extensive and more complete chromosomal characterization of the two living species of Matamatas with a gap of almost half a century since their first karyotype description. Our data surely brings important novelties and shed light on the evolutionary history and adds new pieces to the puzzle of chromosomal evolution in Chelidae. We discovery modes of sex determination and a *mic* XY sex chromosome system, often associated with particular SSR motifs, highlighting ancestry among species that probably dates back to the origin of chelids in the Upper Jurassic ~ 103–160 Mya (Fig. 10). Despite the very similar SSR landscape between the two Matamatas species, such repeats likely drove the pathways that resulted in the different karyotype configurations and sex chromosomes in South America and Australasian lineages. This study is part of a series on cytogenetics and cytogenomics investigation in Amazonian reptiles and their hidden evolutionary diversity.

## Materials and methods

### Sampling, mitotic chromosomes preparation and C-banding

The turtles were sampled from natural populations throughout their distribution, *Chelus orinocensis* in Negro River and *Chelus fimbriata* in Amazonas River. The collects were performed under permission granted by Instituto Chico Mendes de Conservação da Biodiversidade (ICMBio) number: 45275. We analyzed 5 males and 4 females of *Chelus orinocensis* and 3 males and 3 females of *Chelus fimbriata*. Chromosomal preparations were cultured from small blood samples at 29 ℃ according to Viana et al.^25^. The C-positive heterochromatin was detected following Sumner^81^.

All experiments were performed in accordance with relevant guidelines and regulations. We declare that all procedures and experimental protocols were approved and performed under the rules of the Ethics Committee of the National Institute of Amazonian Research (Permission number: 018/2017). The study was carried out in compliance with the ARRIVE guidelines.

### Probes for chromosome hybridization

The 18S rDNA and Telomeric (TTAGGG)n probes were isolated following Gross et al.^82^ and Ijdo et al.^83^, respectively. Both probes were labeled with Aminoallyl-dUTP ATTO-488 (green) by Nick-translation means (Jena Bioscience, Jena, Germany). Simple Short Repeats (SSRs), (AC)_15_, (AT)_15_, (GT)_15_, (AG)_15,_ (AGC)_10,_ (AAT)_10,_ (CGG)_10,_ (AAC)_10,_ (GATA)_8_, (GACA)_8_, (ACGC)_8_, (GGAT)_8_, (AATG)_8_, (AAGG)_8,_ (ATCC)_8,_ (AATC)_8,_ (AAAC)_8_ and (AAAAT)_8_ were used directly labeled with *Cy-3* during the synthesis.

### Fluorescence in situ hybridization (FISH) for repetitive DNA mapping

*FISH* followed the procedures detailed in our previous works^28,84,85^. Briefly, the chromosomes were denatured in 70% formamide/2xSSC at 70 °C, spreads were dehydrated in ethanol (100%). Then, 20 µl of the hybridization mixture (100 ng of each probe, 50% deionized formamide and 10% dextran sulfate) was dropped on the slides and the hybridization was carried out for 24 h at 37 °C in a moist chamber. The chromosomes were counterstained with DAPI (1.2 µg/ml) and mounted in antifade solution (Vector, Burlingame, CA, USA).

### Preparation of probes for comparative genomic hybridization (CGH)

The gDNAs of males and females of Matamatas (*Chelus orinocensis* and *Chelus fimbriata*) were extracted from small blood samples using the Wizard Genomic Purification Kit (Promega), following the manufacturer’s recommendations. Female-derived gDNA was labeled with Aminoallyl-dUTP ATTO-550 (red) and male’ gDNA with Aminoallyl-dUTP ATTO-488 (green) using Nick-translation labeling Kit (Jena Bioscience, Jena, Germany). The final hybridization mixture for each slide (20 μl) was composed of male- and female-derived gDNAs (500 ng each), 25 μg of male-derived Cot-1 DNA (i.e. the fraction of genomic DNA enriched for highly repetitive sequences), prepared following^86^ and the hybridization buffer containing 50% formamide, 2 × SSC, 10% SDS, 10% dextran sulfate and Denhardt´s buffer, pH 7.0. The probes were ethanol-precipitated and the dried pellets were resuspended in hybridization buffer, as above mentioned.

### Comparative Genomic Hybridization (CGH)

The intraespecific CGH experiments were performed according to our previous studies^28,38,85,87^. The slides were incubated at 37° C in a dark humid chamber for three days. The chromosomes were counterstained with DAPI (1.2 µg/ml) and mounted in an antifade solution (Vector, Burlingame, CA, USA).

### Microscopic analyses

At least 10 metaphase spreads for male and females were analyzed to confirm the karyotype structure and *FISH* results. Images were captured using an Olympus BX51 microscope (Olympus Corporation). Chromosomes were classified as macrochromosomes (*Mac*) and microchromosomes (*mic*) or as metacentric (m), submetacentric (sm), subtelocentric (st), and acrocentric (a), according to Levan et al.^88^.

### Ethics statement

All experiments were performed in accordance with relevant guidelines and regulations. We declare that all procedures and experimental protocols were approved and performed under the rules of the Ethics Committee of the National Institute of Amazonian Research (Permission number: 018/2017). The study was carried out in compliance with the ARRIVE guidelines.

## Data Availability

All data generated and analyzed during this study are included in this published article. No datasets were generated during the current study. Additional information about this study is available from the corresponding author upon reasonable request.
